# Cloud Computing Image Processing Application in Athlete Training High-Resolution Image Detection

**DOI:** 10.1155/2022/7423411

**Published:** 2022-10-03

**Authors:** Hongtao Li

**Affiliations:** Physical Education College of Xiangnan University, Chenzhou, Hunan 423000, China

## Abstract

The rapid development of Internet of things mobile application technology and artificial intelligence technology has given birth to a lot of services that can meet the needs of modern life, such as augmented reality technology, face recognition services, and language recognition and translation, which are often applied to various fields, and some other aspects of information communication and processing services. It has been used on various mobile phone, computer, or tablet user clients. Terminal equipment is subject to the ultralow latency and low energy consumption requirements of the above-mentioned applications. Therefore, the gap between resource-demanding application services and resource-limited mobile devices will bring great problems to the current and future development of IoT mobile applications. Based on the local image features of depth images, this paper designs an image detection method for athletes' motion posture. First, according to the characteristics of the local image, the depth image of the athlete obtained through Kinect is converted into bone point data. Next, a 3-stage exploration algorithm is used to perform block matching calculations on the athlete's bone point image to predict the athlete's movement posture. At the same time, using the characteristics of the Euclidean distance of the bone point image, the movement behavior is recognized. According to the experimental results, for some external environmental factors, such as sun illumination and other factors, the image detection method designed in this paper can effectively avoid their interference and influence and show the movement posture of athletes, showing excellent accuracy and robustness in predicting the movement posture of athletes and action recognition. This method can simplify a series of calibration tasks in the initial stage of 3D video surveillance and infer the posture of the observation target and recognize it in real time. The one that has good application values has specific reference values for the same job.

## 1. Introduction

The proposal of mobile cloud computing (MCC) provides new solutions to application requirements [1]. MCC uses the wireless network to offload the application tasks of the mobile terminal to the cloud with stronger computing power for execution, which can solve the problem of insufficient terminal computing power and reduce terminal energy consumption [2]. The cloud server position relative to the location of the user terminal, compared with the traditional server further, rather than the location of the user key network is usually far away, so if we want to realize the customer terminal and exchange data with the cloud server, data transmission can take place not only in the process of extension of time but also in the process of data exchange and resource transmission [3] with the increase of energy consumption. Moreover, for many specific applications, such as voice recognition and intelligent environment control, a long time delay will damage the user experience and affect the performance of related applications.

In order to solve the above shortcomings of mobile cloud computing, the European Telecommunications Standards Institute proposed a new type of computing and storage service technology called mobile edge computing (MEC). As a core technology of 5G, MEC can more effectively address users' needs for specific application services, such as high computing power, storage, reliability, and mobility and low latency, by deploying servers at edge locations closer to users, such as nearby gateways and base stations [4]. The MEC network is used to migrate the computing tasks of geographically dispersed users from mobile terminal devices to resource-rich MEC servers, thereby speeding up the execution of tasks. However, due to the limitation of the communication capacity of the mobile edge computing network and computing resources and the storage capacity of the MEC server, if too many computing tasks are offloaded at the edge, the limited computing resources will bring huge burden to the MEC servers and the user expansion of network transmission and edge computing services expansion of edge computing services [5]. The athlete's motion posture tracking estimation and action recognition method based on the local image features of the athlete's bone points can effectively represent the athlete's motion posture [6]. If the 3D depth video capture technology is used in the experiment to capture the athletes' motion data, the depth image of the movement can be obtained, and the influence of the external environment and other lighting on the experimental results can be avoided to a certain extent so that the accuracy of athletes' motion image detection can be achieved, and more accurate and effective recognition effect can be obtained [7].

## 2. Related Work

Kuang et al. studied the problem of multiuser computing offloading in a dynamic environment. Taking into account the channel interference problem when multiple IoT devices offload computing tasks through the wireless channel at the same time, this research formulates the user's computing offloading decision problem as an evolutionary game model and designs an evolutionary game algorithm based on reinforcement learning used to solve the user's unloading decision [8]. Yuan et al. studied the problem of computing offloading based on the vehicle edge computing network. Considering that the vehicle needs to determine its task offloading strategy in real time in a dynamic network environment, this research proposes a multiuser noncooperative computing offloading game to adjust the task offload probability of each vehicle in the vehicle edge computing network and consider the vehicle at the same time, , while taking into account the distance from the vehicle to the edge computing access point. Furthermore, the research constructed a distributed optimal response algorithm based on the computational unloading game model to maximize the utility of each vehicle [9]. Chen et al. constructed the user unloading problem under the three-tier architecture suitable for mobile and computing scenarios and proposed a distributed balanced computing algorithm to determine the user's computing task unloading decision [10]. Lin et al. studied the problem of multiuser computing offloading of mobile cloud computing in a dynamic environment [11]. Taking into account the problems in the user's free calculation process, it is finally determined that the user's personal privacy rights are settled. Zhang et al. proposed the joint offloading optimization problem of mobile edge computing and cloud computing and designed offloading scheduling and load balancing schemes based on game theory. However, this survey only models and optimizes users' offloading decisions under a hierarchical framework. Edge/cloud computing and communication resource allocation have not yet been optimized [12]. The above-mentioned related studies have given some solutions to the user task offloading decision-making problem, but these studies mainly focus on the user's offloading decision-making problem, ignoring the limited communication and computing resource allocation of the system during the offloading process [13]. In view of the problems in the above research, Yousafzai et al. studied the problem of computing offloading of multiple channels and multiple users under the wireless channel interference environment based on mobile edge-cloud computing, and we jointly studied the problem of computing cloud resource allocation [14]. In order to minimize the energy consumption of users' mobile devices in mobile edge computing systems, Ren et al. proposed a universally available corresponding optimization strategy, which can be applied in computing offloading calculation, resource allocation, and subcarrier allocation [15]. The limited score algorithm has been modified to find the best global solution. The literature proposed a scheme of partial offloading and resource allocation of common user computing tasks, in order to minimize the latency caused by all executions; the required energy consumption, partial offloading, and resource allocation constraints are also investigated. In order to minimize energy consumption and delay, Larson et al. proposed a three-step algorithm: jointly optimizing the offloading decision, calculating user tasks, and allocating communication resources, consisting of semideterministic relaxation, alternate optimization, and continuous adjustment, generated by performing tasks for all users [16]. In this paper, the cost and operation delay of user computing offloading for mobile edge computing are analyzed in depth. In order to reduce the cost of mobile device system and the delay caused by execution as much as possible, this study proposes a multifunctional computing offloading and resource allocation algorithm to jointly optimize the offloading determination and resource allocation of edge users [17]. Reference is oriented to the computing tasks of fog nodes in the industrial Internet of things scenario, with the goal of minimizing energy consumption of fog nodes, so an energy-saving computing offloading scheme is proposed, with comprehensive consideration of fog node energy consumption, local computing, transmission status, and energy consumption in the waiting state [18]. Aiming at the problem of minimizing energy consumption, the research proposed an accelerated gradient algorithm that can quickly find the optimal task offload ratio, thereby improving the convergence speed of the traditional method. This paper designs an algorithm framework that can realize energy-saving offloading. This framework is based on mobile edge computing, which can not only optimize wireless communication resources but also improve the performance of computing resources to a large extent. In addition, in order to reduce the energy consumption of the user system as far as possible, based on the greedy algorithm with the Gini coefficient as a variable, the use of computing resources and operation speed are improved, and an intelligent algorithm system is developed that can greatly reduce the energy consumption of the user when using the client without increasing other consumption. At the same time, this paper also studies the MEC energy-saving calculation program based on a 5G heterogeneous network and its corresponding offloading mechanism. The energy consumption of the system is controlled by jointly optimizing the task unloading program and the wireless resource allocation structure. The 5G network has a unique structural feature; its multiple access can meet the new information transmission form, and combined with the unique nature of this network structure and based on the study of the relevant network transmission structure, this paper designs an energy-saving algorithm, namely, energy-efficient computing and offloading (EECO) algorithm. The literature studied the problem of joint user offloading decision-making and resource optimization at the edge or near-end cloud and proposed a heuristic offloading decision algorithm to improve the utility of the system. However, the above research only considers that user tasks are only offloaded to the edge or the cloud and does not consider the strategy of how users perform offloading and the optimization of resources in the environment of edge-cloud joint computing.

## 3. Research on Cloud Collaborative Multiuser Computing Task Migration

### 3.1. Local Calculation

This article defines the set of the number of users covered by MeNB as *U* *=* {1,2,...,*U*}, where *uU* represents a certain user in the user set. Each user can choose one of the following task execution modes: local computing, which uses local terminal equipment to process tasks; edge computing, in which after offloading tasks to MeNB through the cellular network the tasks will be processed by the edge computing server; and cloud computing, in which tasks are offloaded to MeNB through the cellular network first and then forwarded to the remote cloud server through the core network. In addition, this paper also defines the decision variables for task offloading.

When the user chooses to offload the task to the edge and the cloud, the total time to complete the task includes (1) the time *t*_*u*_^*up*(*e*)^ required for the user to upload the computing task to MeNB, (2) the execution time *t*_*u*_^*exe*(*e*)^ of the user task in MEC, and (3) the time to transmit the result of task completion from MEC to the user equipment. It is assumed that tasks can be unloaded and uploaded to the cloud for a series of uninstallation operations. The time required to upload tasks to the total server is the total time consumed by the whole task process from the start to the end. Considering the downlink transmission, overall, the output of the task is very small compared to the input size of the task.

The speed is much higher than the uplink speed, so this article ignores the time for the task to be transmitted from the cloud to MEC and the time for MEC to be transmitted to the user equipment. This article considers that the user network is a multiuser orthogonal frequency division multiple access system when uploading. Each channel in the system is orthogonal, so the interference in the cell can be ignored. Define *B* as the uplink bandwidth of the wireless link of the system, then the available uplink bandwidth *W*=*B*/*N* for each user, where *N* is the number of users in the cell. Thus, the uplink transmission rate of task *T*_*u*_ for user *u* is

(1) $$\begin{array}{c} R_{u}\left(p_{u}\right)=Wlb\left(1+\frac{p_{u}h_{u}}{\sigma_{0}^{2}}\right)\text{,} \end{array}$$

where *p*_*u*_ represents the transmit power when the input of the upload task of user *u* is *d*_*u*_, which is a positive number and does not exceed the maximum allowed value *P*_*u*_, namely, 0 < *p*_*u*_ ≤ *P*_*u*_; *h*_*u*_ represents the cellular uplink channel gain between user *u* and the base station; and *σ*_0_^2^ represents the transmission background noise power. According to formula (1), the uplink transmission time *t*_*u*_^*up*(*e*)^ for the user *u* to unload the task *T*_*u*_ to MeNB is

(2) $$\begin{array}{c} t_{u}^{up\left(e\right)}=\frac{d_{u}}{R_{u}}\text{.} \end{array}$$

Next, it is necessary to calculate the program runtime spent by user *U* to perform the uninstall task in MEC. This article represents the highest degree limit of computing resources for MEC servers, that is, the number of CPU cycles available in all edge servers. For all end users who issue requests and need to unload tasks and transfer them to the MEC server for computing, they can obtain the computing resources of the MEC server to run the resources in the cloud and share them with the uninstaller. *fe^u^* is defined as the size of the computing resource allocated to the user u offloaded to the edge through the MEC server and must be greater than 0, that is, *fe^u^*>0. However, the computing resources of MEC server are not unlimited, but the highest limit of computing. Therefore, when the actual unloading of resources occurs, the constraints need to be met. When the system is uninstalling, it cannot complete all tasks, and there is always a certain limit., that is, the upper limit of computing resources of the cloud server that needs to be lower than that of the MEC server, which can be expressed as follows:

(3) $$\begin{array}{c} \underset{u\in u_{e}}{\sum }f_{u}^{e}\leq f^{e}\text{.} \end{array}$$

According to the calculation resource size *f*_*u*_^*e*^ allocated by the MEC server, it can be concluded that the calculation time *t*_*u*_^*exe*(*e*)^ of task *T*_*u*_ on the MEC server is

(4) $$\begin{array}{c} t_{u}^{exe\left(e\right)}=\frac{C_{u}}{f_{u}^{e}}\text{.} \end{array}$$

According to formulae (3) and (4), it can be concluded that when the user transmit power *p*_*u*_ is given, the total delay for the user to select the edge computing mode to perform task offloading is

(5) $$\begin{array}{c} t_{u=t_{u}^{up\left(e\right)}+t_{u}^{exe\left(e\right)}}^{e}\text{.} \end{array}$$

The energy consumption *E*_*u*_^*e*^ generated by the user through the margin calculation mode is

(6) $$\begin{array}{c} E_{u}^{e}=p_{u}\frac{d_{u}}{R_{u}}\text{.} \end{array}$$

### 3.2. Cloud Computing

When the user selects the cloud computing mode to perform task offloading, it is assumed that the size of computing resources allocated by the cloud to the offloading task is *f*_*u*_^*c*^. Although the cloud has very rich computing resources, because the number of task requests that require remote cloud computing is very large, the cloud will allocate fixed and limited computing resources to each user. This article assumes that *f*_*u*_^*c*^ is fixed and is equal to the maximum value of computing resources that the cloud can allocate to users. Therefore, similar to formula (6), the computing time *t*_*u*_^*exe*(*c*)^ of the user task in the cloud can be obtained as

(7) $$\begin{array}{c} t_{u}^{exe\left(c\right)}=\frac{C_{u}}{f_{u}^{c}}\text{.} \end{array}$$

Considering that the execution of user tasks in the cloud needs to be transmitted to the remote cloud server through the core network, it can be concluded that the total upload delay of task uninstallation when the cloud execution mode is selected is

(8) $$\begin{array}{c} t_{u}^{up\left(c\right)}=t_{u}^{up\left(e\right)}+t_{u}^{up\left(ec\right)}\\ =\frac{d_{u}}{R_{u}}+\frac{d_{u}}{R_{u}^{c}}\text{,} \end{array}$$

where *t*_*u*_^*up*(*e*)^ is the time for the task to be offloaded from the user equipment to MeNB, *t*_*u*_^*up*(*ec*)^ is the time for the task to be transmitted from MeNB to the cloud, and *t*_*u*_^*up*(*ec*)^ is the transmission rate allocated by the core network to the user *u*. Considering that the total transmission bandwidth of the core network is limited, *R*_*u*_^*c*^ satisfies the constraints shown in the following:

(9) $$\begin{array}{c} \underset{u\in u_{c}}{\sum }R_{u}^{c}\leq R^{c}\text{.} \end{array}$$

According to formulae (8) and (9), it can be concluded that the total delay for users to choose the cloud computing mode to perform task offloading is

(10) $$\begin{array}{c} t_{u}^{c}=t_{u}^{up\left(c\right)}+t_{u}^{exe\left(c\right)}\text{.} \end{array}$$

Since the user only consumes energy when uploading tasks to MeNB, the energy consumption generated by the user through the cloud computing mode is

(11) $$\begin{array}{c} E_{u}^{c}=E_{u}^{e}\\ =p_{u}\frac{d_{u}}{R_{u}}\text{.} \end{array}$$

### 3.3. System Utility Maximization Problem Based on Edge-Cloud Joint Computing

Under the edge-cloud joint computing framework, the user's QoE is mainly reflected by the delay and energy consumption in completing tasks. Based on the above content, the uninstallation model and user preferences can be calculated in each mode. This article defines the uninstallation utility function using *u* as

(12) $$\begin{array}{c} V_{u}=x_{u,1}\left(\beta_{u}^{t}\frac{t_{u}^{l}-t_{u}^{e}}{t_{u}^{l}}+\beta_{u}^{e}\frac{E_{u}^{l}-E_{u}^{e}}{E_{u}^{l}}\right)+x_{u,2}\left(\beta_{u}^{e}\frac{t_{u}^{l}-t_{u}^{ce}}{t_{u}^{l}}+\beta_{u}^{e}\frac{E_{u}^{l}-E_{u}^{c}}{E_{u}^{l}}\right)\text{,} \end{array}$$

where *β*_*u*_^*t*^ and *β*_*u*_^*e*^, respectively, represent the user's preference weight for the time delay and energy consumption to complete the task and *β*_*u*_^*e*^, *β*_*u*_^*t*^ ∈ [0,1], *β*_*u*_^*e*^+*β*_*u*_^*t*^=1. For example, when the user *u*'s device battery can be used for a short period of time, the user will prefer to increase the value of *β*_*u*_^*e*^ to save power at the expense of time delay. Based on the above-mentioned unloading utility function of user *u*, this paper defines the system utility function as *V*=∑_*u*=1_^*U*^*V*_*u*_.

The above-mentioned system utility function model involves the allocation of communication resources, edge server computing resources, and cloud transmission resources. It not only considers the utility of users but also pays attention to the resource allocation of resource providers. Therefore, this paper expresses the system utility maximization problem based on edge-cloud joint computing as

(13) $$\begin{array}{c} \begin{array}{c} \begin{array}{c} \begin{array}{c} \max\\ f,x,p,R \end{array}V=\overset{U}{\underset{u=1}{\sum }}V_{u}\\ s.t.C1:x_{u,j}=\left\{0,1\right\},u\in U,j\in \left\{1,2\right\},\\ C2:0<p_{u}\leq P_{u},\forall_{u}\in U, \end{array}\\ C3:\underset{u\in U_{e}}{\sum }f_{u}^{e}\leq f^{e},\\ C4:f_{u}^{e}>0,\forall_{u}\in U_{e}, \end{array}\\ C5:\underset{u\in U_{c}}{\sum }R_{u}^{c}\leq R^{c}\text{,}\\ C6:R_{u}^{c}>0,\forall_{u}\in U_{c}\text{.} \end{array}$$

By optimizing the offloading decision *X*, and optimizing the communication resources and computing resources at the same time, the synergistic effect of the two can be achieved, and the peak value problem of the above system utility can be solved. Since the unloading decision *x* is a 0-1 integer vector and *f*, *p*, and *R* are continuous vectors, the optimization problem of formula (14) is an MINLP. Considering the expression structure of the optimization problem, when the value of the unloading decision *x* is given, the original optimization problem with higher complexity can be decomposed into the main problem with lower complexity and a series of subproblems. Therefore, the problem shown in formula (13) can be transformed into

(14) $$\begin{array}{c} \begin{array}{c} \max\\ x\\ \max\\ R,f,p \end{array}\underset{u\in U_{e}\cup U_{c}}{\sum }V_{u}s.t.\;C1∼C6\text{.} \end{array}$$

Since the constraints *C*1 of unloading decision and *C*2–*C*6 of resource allocation policy are separable, the optimization problem shown in formula (14) can be decomposed into the main problem and subproblem, as shown in the following formulae, respectively:

(15) $$\begin{array}{c} \begin{array}{c} \max\;V^{*}\\ x \end{array}s.t.C1\text{,} \end{array}$$

(16) $$\begin{array}{c} V^{*}=\begin{array}{c} \max\\ R,f,p \end{array}\underset{u\in U_{e}\cup U_{c}}{\sum }V_{u}s.t.\;C2∼C6\text{.} \end{array}$$

Decomposing the optimization problem of formula (14) into the optimization problems of formulae (15) and (16) does not change its optimal solution. Next, this article will give the optimization problems of formulae (15) and (16), respectively, and finally solve the problem of formula (17).

### 3.4. Joint Optimization of the Edge-Cloud Resource Method

According to the form of formula (13), when the unloading decision *x* is given, the optimization problem of formula (17) can be transformed into

(17) $$\begin{array}{c} \begin{array}{c} \max\;V\\ R,f,p \end{array}\left(x,p,f,R\right)=\begin{array}{c} \max\;V\\ R,f,p \end{array}\underset{u\in U_{e}\cup U_{c}}{\sum }\left(\beta_{u}^{t}+\beta_{u}^{e}\right)-I\left(x,p,f,R\right)\text{,} \end{array}$$

where *I*(*x*, *p*, *f*, *R*) is

(18) $$\begin{array}{c} I\left(x,p,f,R\right)=\underset{u\in U_{e}}{\sum }\left(\beta_{u}^{t}\frac{t_{u}^{e}}{t_{u}^{l}}+\beta_{u}^{e}\frac{E_{u}^{e}}{E_{u}^{l}}\right)+\underset{u\in U_{e}}{\sum }\left(\beta_{u}^{t}\frac{t_{u}^{c}}{t_{u}^{l}}+\beta_{u}^{e}\frac{E_{u}^{c}}{E_{u}^{l}}\right)\text{.} \end{array}$$

When the unloading decision *x* is given, ∑_*u*∈*U*_*e*_∪*U*_*c*__(*β*_*u*_^*t*^+*β*_*u*_^*e*^) is a constant, so formula (18) can be transformed into an *I*(*x*, *p*, *f*, *R*) minimization problem, namely,

(19) $$\begin{array}{c} \begin{array}{c} \min\;I\\ p,f,R \end{array}\left(x,p,f,R\right)s.t.\;C2∼C6\text{.} \end{array}$$

According to formulae (1)∼(19), we can get

(20) $$\begin{array}{c} I\left(x,p,f,R\right)=\underset{u\in U_{e}\cup U_{c}}{\sum }\frac{\emptyset_{u}+\varphi_{u}p_{u}}{lb\left(1+p_{u}\gamma_{u}\right)}+\underset{u\in U_{e}}{\sum }\frac{\beta_{u}^{t}f_{u}^{l}}{f_{u}^{e}}+\underset{u\in U_{c}}{\sum }\frac{\beta_{u}^{t}f_{u}^{l}}{f_{u}^{c}}+\underset{u\in U_{c}}{\sum }\frac{\beta_{u}^{t}d_{u}f_{u}^{l}}{c_{u}R_{u}^{c}}\text{,} \end{array}$$

where ∅_*u*_=*β*_*u*_^*t*^*d*_*u*_/*t*_*u*_^*l*^*W*, *φ*_*u*_=*β*_*u*_^*t*^*d*_*u*_/*E*_*u*_^*l*^*W*, and *γ*_*u*_=*h*_*u*_/*σ*_0_.

According to the form of formula (20), it can be found that when the uninstallation strategy *x* is given, the third term on the right side of the equal sign in formula (20) is a constant.

### 3.5. Analysis of Simulation Results

The *P*_*u*_^*l*^ corresponding to the computing power of the user equipment selected in this paper is {0.5 W, 0.75 W, 0.9 W}. If the user's maximum transmission power *P*_*u*_ = 100 mW, the total uplink transmission rate from MeNB to the cloud is *R*^*c*^ = 100Mbit/s. Other relevant simulation parameter settings are shown in Table 1.

Under the same parameter settings, this section chooses to compare the system utility performance of the user offloading strategy based on the edge-cloud joint computing solution proposed in this paper with the following solutions:

1. Local computing: all users use local computing to complete tasks.
2. Total offloading strategy of joint resource optimization based on edge computing: all users offload tasks to the edge for execution and adopt an optimized resource allocation plan.
3. Total offloading strategy of joint resource optimization based on cloud computing: all users offload tasks to the cloud for execution and adopt an optimized resource allocation plan.

Without loss of generality, the simulation results are the results of each simulation experiment repeated 1,000 times and averaged.

**Figure 1:**
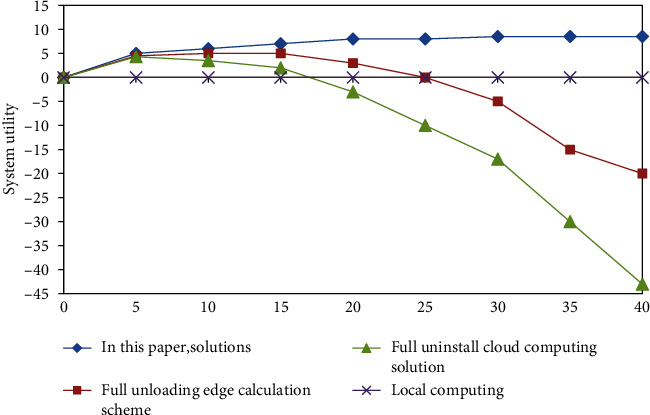
System utility of each scheme under different total numbers of users.

The system utility value of each program will change with the change of the number of users, and the trend of each program is shown in Figure 1. The overall observation and analysis of Figure 1 shows that the system utility value of the solution designed in this paper has made great progress and is relatively stable when compared with that of other similar schemes under various states with the increase of the number of users. Except for the local calculation scheme, when the number of users is at a low level, the relationship between the system utility value of the other three schemes and the number of users is in direct proportion, and the system utility value of the solution in this paper is better than that of the other two schemes. The graph shows that the system utility value of the local calculation scheme is always 0. And when the number of users is once more than a given value, the system running will reach a peak, the increase of users in the cloud computing scheme of the system is inversely proportional to the utility value roughly, which already cannot satisfy the user's demand to uninstall the program, and the utility value of the other two calculation schemes is lower than that of the local calculation scheme and decreases with the increase of the number of users, so there is an inverse relationship. Compared with that of the local calculation scheme, the system utility value of the solution in this paper is always at a higher stable level in the process of increasing the number of users. This is because when the number of offloaded users is too large, the limited uplink communication resources and edge computing resources cannot provide richer resource requirements for each demanding user, resulting in higher computing time and transmission delay. Users sending and executing tasks in a fully uninstalled manner will cause all users to compete for limited resources. When there are too many unloaded users, the computing resources allocated to users by edge nodes under the edge computing solution will be lower than the local computing resources, and the low uplink communication resources of users under the cloud computing solution will cause the transmission delay of task offloading to be too high. In turn, the system utility of the above two schemes is reduced or even lower than the utility of local computing. With the increase of users, the user uninstallation strategy selection algorithm under this scheme can still maintain a high and stable system utility value. This is because the solution can reasonably plan the mode selection of task offloading for users, thereby ensuring the maximum utilization of limited computing and communication resources.

**Figure 2:**
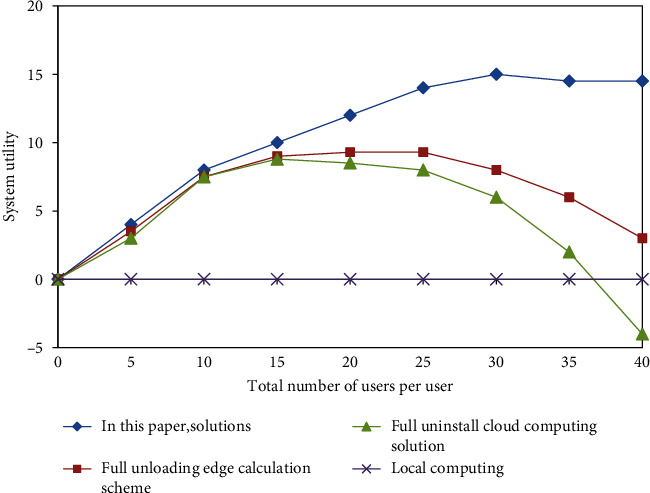
System utility of each scheme under the condition of different total users under specific tasks.

In this paper, the actual scenario of face recognition application of this system is selected, and the system utility performance of the proposed solution is used to conduct experiments and evaluations on index changes. Firstly, in order to complete the task, the total amount of computing resources required by the cloud is Cu = 1000 Mcycle. In addition, when data are input, their size is du = 420 KB, and the experimental results are shown in Figure 2.

From the observation and analysis of Figure 2, it can be concluded that the system utility value of the proposed solution performs significantly better than the numerical display of the other two solutions in the specific application task of face recognition, and it always maintains a certain level of efficiency. In addition, with the increase of the number of users, the system utility value of the other two schemes will decrease by different magnitudes after reaching a certain threshold upper limit, while the system utility of the solution proposed in this paper can still maintain a high and stable running state. The solution in this paper combines all available computing and communication resources on the edge, cloud, and local sides to maintain a stable and dominant system utility when the number of users increases.

## 4. Application of High-Resolution Image Detection for Athlete Training Based on Visual Images

### 4.1. Athlete Motion Posture Tracking Estimation and Recognition Method Based on Visual Images

Now, methods for obtaining 3D depth images of athletes can be divided into two categories: methods based on athlete boundary objects and methods based on depth cameras. The former requires multiple testing devices and sensors on the observation object. Although the boundary-based identification method has high sensitivity and accuracy, it is not suitable for general research because of its high cost and very complex operation. With the improvement of the technical level, the depth camera-based modeling method is developing very rapidly. In the aspect of 3D depth image acquisition, it has been gradually applied as the main method.

**Figure 3:**
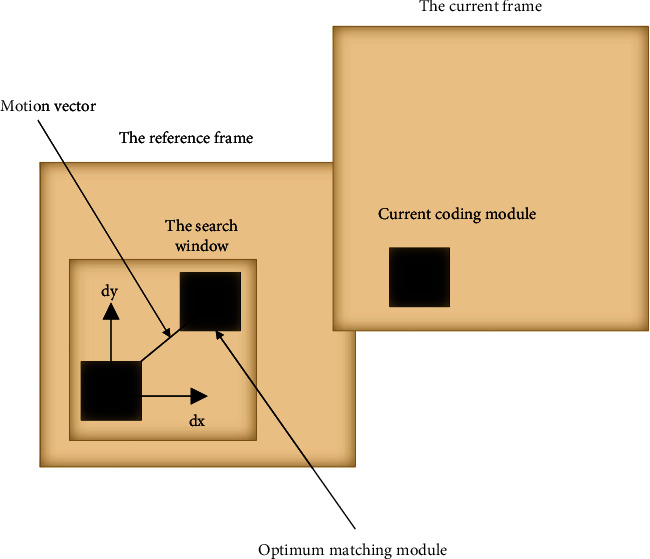
Schematic diagram of the block matching method.

In this paper, a 3-stage exploration algorithm TSS based on the block matching method is used to track and estimate the motion pose of the target. The block matching method is a motion estimation algorithm based on local image features. Set the target macroblock in the observation image, find the best motion direction of the target macroblock between adjacent image frames, calculate the motion displacement vector, and perform motion estimation of the observation target. The schematic diagram of the block matching method is shown in Figure 3.

High precision and high speed are the basis for real-time tracking and estimation of athletes' motion posture in the athlete's motion posture detection method. Based on the local image characteristics of the target, the three-step search algorithm finds the smallest absolute value difference between macroblocks according to the search range from large to small patterns.

The block matching algorithm of the three-step search algorithm applies the minimum average absolute value difference method for calculation, and the calculation method is shown as

(21) $$\begin{array}{c} MAD\left(d_{x},d_{y}\right)=\frac{1}{MN}\underset{\left(x_{1},y_{1}\right)\in B}{\sum }\left|f_{k}\left(x_{1},y_{1}\right)-f_{k-1}\left(x_{1}+d_{x},y_{1}+d_{y}\right)\right|\text{.} \end{array}$$

**Figure 4:**
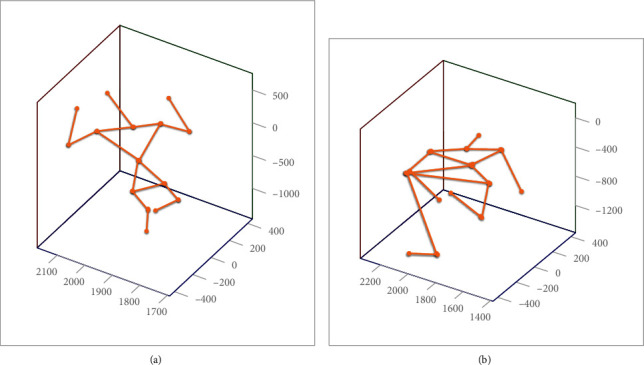
Example diagram of the athlete's 3D skeleton point posture. (a) Sample image of standard posture. (b) Image action in the process of motion.

In the aspect of the action recognition of athletes, this paper mainly designs an action recognition method based on the local image features of the athlete's skeleton.  Figure 4 is a 3D coordinate diagram of the athlete's bone points obtained by the Kinect depth camera. After observing the target extinction, the local coordinates of the athlete's bone point data and altitude will change.

After obtaining the performance characteristics of the athlete's sports behavior, this paper uses the Euclidean distance to calculate the displacement *D*. The athlete's bone point image does not need to consider the three-dimensional coordinates of the camera to detect the local characteristics of the adjacent time, the image bone point, and the *z*-coordinate target distance. The calculation formula of displacement *D* is shown as

(22) $$\begin{array}{c} D=\sqrt{{\left(x_{i}-y_{i}\right)}^{2}+{\left(x_{j}-y_{j}\right)}^{2}}\text{,} \end{array}$$

which represents the relative change of the local features of the athlete in the skeleton point image to identify the athlete's movement. According to experience, a threshold value is set for the local characteristic changes of the bone points of the destrength attack action, and *D* is obtained, which is compared with the threshold value. When *D* is greater than the set threshold, it is decided to act as a destrength attack. Otherwise, it is judged that the operation is normal. The example diagram of the athlete's 3D skeleton point posture is shown in Figure 4.

### 4.2. High-Resolution Image Detection Algorithm

The KSVD algorithm is a powerful tool for sparse scoring. *D_x_* represents dictionary-based information. KSVD can be used in combination with various tracking algorithms. The following formula is used to express the objective function of solving the coefficient:

(23) $$\begin{array}{c} \begin{array}{c} \min\\ x \end{array}{\left\|x\right\|}_{0}s.t.y=Dx\text{,}\\ \begin{array}{c} \min\\ x \end{array}{\left\|x\right\|}_{0}s.t.\left\|y-Dx\right\|\leq \varepsilon \text{.} \end{array}$$

In the face of high-resolution SAR images, the detailed information of the SAR image will increase significantly, and the complexity of the object will also increase. At the same time, high-resolution SAR images will also lose the general SAR image texture function. In the same area, the distribution parameters are also different (because of the large amount of gradient information, the traditional resolution SAR image information does not exist, but the high-resolution SAR image information obviously exists), but they are used to create more refined and clear partitions. All these models can only describe areas with a few simple targets and terrain types. In other words, statistical models have “regional characteristics.” In a large-area scene, because the target and terrain are complex, it is not practical to use only a statistical model containing a few parameters to describe the entire image. However, if there are too many parameters, the application may have problems. If we want to train a dictionary for these broad characteristics, the dimension of the dictionary will inevitably become excessive. Moreover, due to insufficient data, the number of features is far greater than the amount of data available for training, so overmatching will inevitably occur.

In order to solve the above problems, we propose a hierarchical dictionary learning method to solve the problem of too high dimensionality of the dictionary when simulating high-resolution SAR images. This algorithm will solve the self-learning dictionary problem in high-resolution SAR image detection. However, due to the large geographical features of the ultrahigh resolution SAR image, the dimensionality of the dictionary is very high, and there are some cases that cannot be tolerated and cannot solve the problem. In this paper, we introduce a hierarchical algorithm, namely, the grammar in a hierarchical structure. According to the size of the target vehicle, high-resolution SAR images are regional characteristics. In this article, in SAR images, since the average vehicle target resolution is above 1000 pixels (0.3 meters), the target and shape will be considered. Shadows themselves also have certain distribution characteristics. Like the idea of classification, they are formed between specific information structures. The distribution characteristics and structure information are used to detect targets and complete incomplete targets.

The basic principle of the hierarchical dictionary learning method is to make dictionaries of each category (target, grass, runway, building, tree, etc.) of different SAR images (the same category of different SAR images also has obvious differences). Moreover, the dimension of the dictionary may reach thousands of dimensions (or 10,000 dimensions). But at the same time, because different SAR images are used as different areas of the dictionary on specific characteristics, and one of the most obvious differences in darkness requires different SAR images, different images can be used to construct the dictionary part of the grayscale average mark and the background area. The calculation ofgray-level average value starts from the image with too many possibilities. The simple distribution parameters obtained from the training image are shown in Table 2.

After constructing the dictionary, the problem to be faced is how to quickly find *α* and *λ* in Ω_∨_=Ω*λ*^*T*^ and *y* = Ω_∨_*α* as features. The solution to this can be transformed into the following equation:

(24) $$\begin{array}{c} \alpha =arg\begin{array}{c} \min\\ y \end{array}{\left\|\Omega_{\wedge }y\right\|}_{0}\\ =arg\begin{array}{c} \min\\ y \end{array}{\left\|\Omega \lambda^{T}y\right\|}_{0}\text{,} \end{array}$$

where Ω is a dictionary and *α* is the coefficient of *x* in the dictionary. The objective function form of the basis tracking (BP) algorithm is

(25) $$\begin{array}{c} \min{\left\|\alpha \right\|}_{1}s.t.A=\Omega \alpha \text{.} \end{array}$$

This algorithm is relatively simple and is not common in practical applications, but it is a very basic method. Understanding this algorithm will help deepen the understanding of subsequent algorithms.

As one of the classic methods of analyzing signal decomposition, the match pursuit (MP) algorithm is widely used, and the effect is remarkable. The purpose is to use a complete dictionary to disassemble the sample, and the expression after decomposition is required to be as correct as possible. Assuming that the sample signal *f* represented belongs to the Hilbert space, *f* is expressed as the weighted sum of several elements selected in the dictionary *D*:

(26) $$\begin{array}{c} f\left(x\right)=\underset{i}{\sum }a_{i}g_{i}\left(x\right),i=1,2,\ldots ,N\text{.} \end{array}$$

If a dictionary is given, the MP algorithm first uses the inner product of the largest original signal correlation (the largest) as the gesture of *f*(*x*) to select, then selects the similarity of the two signals as the corresponding weight, and then selects the larger signal. Combine the *f*(*x*) of the above tuple until the inner product of the elements and the selected signal better fit the edge of *f*(*x*). The steps of the MP algorithm are shown in Table 3.

The MP algorithm is often used in the fields of signal coding, image coding, video coding, etc., to obtain better tracking results. This algorithm has some limitations, such as high computational complexity and low ease of use in applications that require real-time tracking.

The orthogonal matching pursuit (OMP) algorithm is based on the extension of MP algorithm. The objective function of this algorithm can be roughly expressed by

(27) $$\begin{array}{c} \min{\left\|f-\Omega \alpha \right\|}_{F}^{2}s.t.{\left\|\alpha \right\|}_{0}\ll N\text{.} \end{array}$$

### 4.3. Athlete Training High-Resolution Image Detection Results and Analysis

Using the initialization conditions proposed in this paper, the orthogonal matching tracking algorithm is used to obtain the characteristic parameter vector of the data, as shown in Table 4.

Through the above test, it is obvious that the tracking algorithm using the overmatching dictionary proposed in this paper can better adapt to the data model. By using this method, the obtained coefficient vector can be used as the feature of subsequent classification and detection steps, and good results can be obtained after testing. In the dictionary tracking process, spactive is used to express features. In other words, there are few elements other than zero in the coefficient vector obtained by tracking. Therefore, the classification and detection process will not cause a lot of time loss. On the contrary, the tracking process does not require a lot of mathematical optimization, so the calculation time can be further reduced.

**Figure 5:**
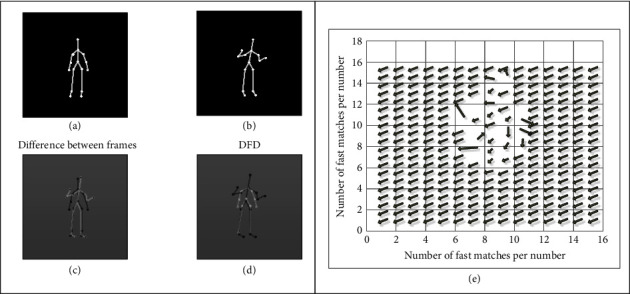
Motion estimation experiment results under sufficient light conditions. (a) Interframe difference of the first frame; (b) DFD value of the second frame; (c) Image difference between the two frames; (d) Difference between the displacement frames; (e) Fast matching of each digit in the second frame image vector.

A three-step search algorithm is used to calculate the displacement vector of the bone point image. After error correction, as shown in Figure 5, the displacement vector diagram of the bone point image of the athlete's movement posture can be obtained. The experimental results show that the robustness of the three-step search algorithm is good. The lighting and the athlete's movement track and direction can be obtained from the displacement vector to estimate the athlete's movement behavior.

## 5. Conclusion

In the detection of sports postures of athletes, because two-dimensional video images cannot overcome the influence of the lighting environment, a real-time image detection method of sports postures of athletes based on the local image features of depth images is proposed. First, a 3D depth image is obtained based on the scene depth camera, and the depth image is converted into an image of the athlete's bone point. In order to achieve the purpose of estimating the movement posture of athletes, it is necessary to calculate the displacement vector of the local features of the bone point image and use the three-step search algorithm to calculate the specific movement direction of athletes. In addition, it is necessary to calculate the Euclidean distance change of the local features of the bone point image and identify the movement behavior of athletes. Through the analysis of the experimental data results, it can be concluded that the method in this paper can accurately detect and obtain the athletes' movement posture and effectively avoid the influence caused by external factors such as light, showing great positive significance for the accuracy of the tracking estimation of athletes' movement posture and athletes' action recognition and simplifying a series of calibration tasks for observed targets. It is the initial stage of the video surveillance process to achieve the effect of real-time surveillance. Athlete posture detection is the basis of athlete tracking estimation and motion recognition, which is widely used in video surveillance technology. It has very important application value in motion recognition monitoring, social network security, social and family monitoring, etc. This dynamic image recognition method based on local image features can help to solve the problem of randomness and nonrigidity of athletes' movement and has certain effectiveness in the expression of athletes' movement behavior. Currently, local image feature detection methods based on two-dimensional images cannot overcome the effects of changes in the lighting environment and cannot detect observation objects in real time. The detection method based on the local image features of the 3D depth image can flexibly measure the three-dimensional information of the target under various conditions and effectively overcome the influence of the light environment. In addition, there is no need to set the calibration object of the observation object, and the posture estimation and action recognition of the object are performed in real time.

## Figures and Tables

**Table 1:** Simulation parameter settings.

Parameter name	Value
System bandwidth B (MHz)	20
Path loss model	128.1 + 37.5lg10(d)
Background noise *σ*_0_^2^ (dBM)	100
Standard deviation of lognormal shadow fading (dB)	10
Edge computing resources *f*^*e*^ (GHz)	20
Computing resources allocated to users in the cloud *f*_*u*_^*c*^ (GHz)	5

**Table 2:** Initialization of model parameters.

Distribution image	Gaussian distribution		Generalized gamma distribution			Fourth-order polynomial distribution
	*μ*	*δ*	*α*	*δ*	*λ*	
Background	69.12	1205.53	0.1260	2.09*E* − 7	28.89	−5.88*E* − 9 4.15*E* − 6 −6.24*E* − 3 7.06*E* − 2 −1.98*E* − 4
Background	58.05	1055.82	0.1338	1.04*E* − 10	36.48	−8.11*E* − 11 4.77*E* − 8 −9.13*E* − 6 5.65*E* − 4 7.56*E* − 4
Dark scene	20.20	122.41	0.1280	1.08*E* − 11	36.48	1.16*E* − 12 −9.10*E* − 9 4.39*E* − 6 −6.80*E* − 4 0.0323
Building	151.87	5210.61	0.3560	0.0852	14.00	7.69*E* − 11 −3.42*E* − 8 4.54*E* − 6 −1.62*E* − 4 0.0026
Shadow	14.39	99.12	0.1671	2.19*E*−8	28.98	1.07*E*−10 −6.93*E*−8 1.58*E*−5 −0.0015 0.0467
Shadow	13.35	42.62	0.2248	8.90*E* − 6	23.97	1.16*E* − 10 −7.46*E* − 8 1.68*E* − 5 −0.0016 0.0483
Aims	183.49	5967.72	0.5564	4.84	7.41	1.68*E* − 10 −7.52*E* − 8 1.05*E* − 5 −4.85*E* − 4 0.0064
Min	13.35	42.62	0.1280	1.08*E* − 11	7.41	1.07*E* − 10 −7.52*E* − 8 −7.64*E* − 6 −0.0016 −2.63*E* − 4
Max	183.49	5967.72	0.5564	4.84	36.48	1.16*E* − 12 4.77*E* − 8 1.68*E* − 5 5.65*E* − 4 0.0483

**Table 3:** MP algorithm description.

Enter	To be represented signal *f*(*x*), dictionary *D*
Output	Tracking results, representing the yuan and its corresponding coefficients *a*_*i*_*g*_*i*_(*x*)
Algorithm initialization	*R*_1_=*f*(*x*)
Cycle strategy	*g*_*i*_(*x*) ∈ *Ds*.*t*. max {|〈*f*(*x*), *g*_*i*_(*x*)〉|} *a*_*i*_=|〈*f*(*x*), *g*_*i*_(*x*)〉|*R*_*i*+1_=*R*_*i*_ − *a*_*i*_*g*_*i*_(*x*)
Termination condition	‖*R*_*i*_‖ < Threshol d

**Table 4:** Fit test.

Distribution/estimation map/assessment		GRD		Gaussian		Method of this paper
		MoM	MoLC	MoM	MoLC	Dictionary
Figure a	KL	0.8101	0.7244	1.0758	0.9125	0.0214
	KS	0.1012	0.3034	0.1419	0.1716	0.0145
	MSE	2.6*e* − 4	2.2*e* − 4	7.7*e* − 3	4.5*e* − 4	1.7*e* − 9

Figure b	KL	0.2318	0.4517	0.9578	1.2651	0.0145
	KS	0.0988	0.817	0.1099	0.0977	0.0020
	MSE	4.3*e* − 3	2.2*e* − 2	3.8*e* − 3	5.5*e* − 2	1.4*e* − 9

Figure c	KL	0.4007	0.1917	0.2381	0.3219	0.0098
	KS	0.1372	0.2418	0.6099	0.4917	0.0124
	MSE	9.8*e* − 7	2.2*e* − 6	5.8*e* − 7	2.2*e* − 6	7.9*e* − 10

Figure d	KL	0.8198	0.2789	0.2237	0.5941	0.0914
	KS	1.0911	1.3412	1.0901	0,9879	0.0038
	MSE	5.7*e* − 4	3.2*e* − 4	5.1*e* − 5	9.7*e* − 6	3.7*e* − 8

Figure e	KL	1.1098	1.0091	0.9178	0.9914	0.0099
	KS	0.7816	0.4648	0.7816	0.8817	0.0014
	MSE	1.4*e* − 5	4.7*e* − 6	8.9*e* − 6	2.6*e* − 5	5.4*e* − 10

Figure f	KL	0.1498	0.1147	0.4512	0.3645	0.0012
	KS	0.1278	0.2918	0.7828	0.6514	0.0009
	MSE	5.4*e* − 10	5.4*e* − 10	5.4*e* − 10	5.4*e* − 10	4.4*e* − 10

## Data Availability

The data used to support the findings of this study are available from the author upon request.
